# Molecular Characterization and Overexpression of *SmJMT* Increases the Production of Phenolic Acids in *Salvia miltiorrhiza*

**DOI:** 10.3390/ijms19123788

**Published:** 2018-11-28

**Authors:** Bin Wang, Junfeng Niu, Bin Li, Yaya Huang, Limin Han, Yuanchu Liu, Wen Zhou, Suying Hu, Lin Li, Donghao Wang, Shiqiang Wang, Xiaoyan Cao, Zhezhi Wang

**Affiliations:** 1 National Engineering Laboratory for Resource Development of Endangered Crude Drugs in Northwest China, Key Laboratory of the Ministry of Education for Medicinal Resources and Natural Pharmaceutical Chemistry, College of Life Sciences, Shaanxi Normal University, Xi’an 710119, China; happywangbin2003@163.com (B.W.); niujunfeng6829@126.com (J.N.); libin1989@snnu.edu.cn (B.L.); h_yoyo@snnu.edu.cn (Y.H.); hdd_1981_@163.com (L.H.); shidayuanchu@snnu.edu.cn (Y.L.); wenzhou0229@snnu.edu.cn (W.Z.); husuying0315@163.com (S.H.); shidalilin@snnu.edu.cn (L.L.); wangdonghao@snnu.edu.cn (D.W.); wsq@snnu.edu.cn (S.W.); 2 College of Chemistry, Biology and Materials Science, East China University of Technology, NanChang 330013, China

**Keywords:** *SmJMT*, transgenic, *Salvia miltiorrhiza*, overexpression, transcriptome, phenolic acids

## Abstract

Jasmonic acid (JA) carboxyl methyltransferase (JMT), a key enzyme in jasmonate-regulated plant responses, may be involved in plant defense and development by methylating JA to MeJA, thus influencing the concentrations of MeJA in plant. In this study, we isolated the *JMT* gene from *Salvia miltiorrhiza*, an important medicinal plant widely used to treat cardiovascular disease. We present a genetic manipulation strategy to enhance the production of phenolic acids by overexpresion *SmJMT* in *S. miltiorrhiza*. Global transcriptomic analysis using RNA sequencing showed that the expression levels of genes involved in the biosynthesis pathway of phenolic acids and MeJA were upregulated in the overexpression lines. In addition, the levels of endogenous MeJA, and the accumulation of rosmarinic acid (RA) and salvianolic acid (Sal B), as well as the concentrations of total phenolics and total flavonoids in transgenic lines, were significantly elevated compared with the untransformed control. Our results demonstrate that overexpression of *SmJMT* promotes the production of phenolic acids through simultaneously activating genes encoding key enzymes involved in the biosynthesis pathway of phenolic acids and enhancing the endogenous MeJA levels in *S. miltiorrhiza*.

## 1. Introduction

*Salvia miltiorrhiza* Bunge (Lamiaceae) is a well-known traditional Chinese herb with significant medicinal and economic value. Its dry roots or rhizomes (called “danshen” in Chinese) are used to treat various cerebrovascular and cardiovascular diseases in Asian countries, and are widely accepted as a health supplement in western countries [1,2]. Due to its remarkable and reliable therapeutic actions, *S. miltiorrhiza* is being developed as a potential model for research into traditional Chinese medicine [3]. Furthermore, by virtue of high-throughput technologies, the genomic sequence of *S. miltiorrhiza* was published [4].

*S. miltiorrhiza* contains two major medicinal components that are largely responsible for the observed pharmacological activities; one is a group of lipid-soluble (non-polar, lipophilic) diterpenoids, known as tanshinones, and the other is a water-soluble (polar, hydrophilic) group of phenolic acids, such as rosmarinic acid (RA) and salvianolic acid B (Sal B) [5]. *S. miltiorrhiza* is traditionally processed through extraction with water. Sal B becomes the predominant active ingredient among the phenolic acids, which is designated as a marker component of *S. miltiorrhiza* in the official Chinese Pharmacopoeia. In additional, it was reported that Sal B can provide protection against cardiovascular, neural, and hepatic diseases, as well as certain cancers [6]. Although beneficial to human health, the concentration of Sal B is low in commercial cultivars of *S. miltiorrhiza*, which currently limits its widespread use and medicinal efficiency. Furthermore, Sal B is difficult to purify from complex mixtures, resulting in inefficient chemical synthesis [7].

In *S. miltiorrhiza*, the biosynthetic pathway leading to Sal B and RA is thought to entail both the phenylpropanoid and tyrosine-derived pathways [7]. Enzymes involved in the phenylpropanoid pathway include phenylalanine ammonia-lyase (PAL), cinnamate 4-hydroxylase (C4H), and 4-coumarate/coenzyme A ligase (4CL). Tyrosine aminotransferase (TAT) and hydroxyphenylpyruvate reductase (HPPR) are active in the tyrosine-derived pathway. An additional enzyme, rosmarinic acid synthase (RAS), couples products from the two pathways. This product is then hydroxylated by cytochrome P450 monooxygenase C3’H (CYP98A) to form RA [8]. Many methods were identified for increasing the production rates of such health-promoting phenolic acids, such as genetic engineering [7], hormone induction [9], and biological fermentation [10]. Jasmonate treatment is the most commonly used method of hormone elicitation.

Jasmonates which include jasmonic acid (JA), methyl jasmonate (MeJA), and its cyclopentane derivatives, are a class of plant hormone that regulate many aspects of plant development such as root growth, production of viable pollen, fruit ripening, and senescence [11,12]. They are also involved in plant responses to biotic and abiotic stresses including insect attack, wounding, water deficiency, ultraviolet (UV) light, pathogen infection, and ozone [13,14,15]. Jasmonates are synthesized in plants via the octadecanoid pathway [14,16] from α-linolenic acid through a series of enzymes, beginning with an oxygenation catalyzed by lipoxygenase (LOX) [17]. The product is then converted to 12-oxo-phytodienoic acid (12-OPDA) by allene oxide synthase (AOS) and alleneoxide cyclase (AOC) [18,19]. Afterward, JA is synthesized from 12-oxo-phytodienoic acid (12-OPDA) through reduction by 12-oxo-phytodienoic acid reductase (OPR) and three steps of β-oxidation, and formation of MeJA is catalyzed by jasmonic acid carboxyl methyltransferase (JMT).

Jasmonic acid carboxyl methyltransferase (JMT) is an *S*-adenosyl-l-methionine-dependent methyltransferase of the SABATH gene family, which could specifically methylate carboxyl groups of small molecules such as jasmonic acid, salicylic acid, and benzoic acid, and named based on the first three identified genes belonging to this family, *SAMT*, *BAMT*, and that coding for theobromine synthase [20]. The *JMT* gene was first identified in *Arabidopsis* [21], and then was successively found in *Capsicum annum* [22], *Populus trichocarpa* [23], strawberry [17], and rice [24]. *JMT* acts as a cellular regulator of the level of physiologically active JA [17,24], which functions in response to several different external environmental stimuli [21] and mediates diverse developmental processes in plants [24,25]. Transgenic *Arabidopsis* lines overexpressing *JMT* had an elevated level of endogenous MeJA, and the transgenic plants showed enhanced levels of resistance against the virulent fungus [21]. Meanwhile, overexpressing *Arabidopsis JMT* in potato increased tuber yield and size [26].

In plants, MeJA, a signal molecule that acts as a second messenger, is proposed to play a role in the elicitation process [27,28], which could lead to the accumulation of secondary metabolites [29]. Furthermore, MeJA could induce plant tissues to provide a responsive system to identify and profile the transcripts and regulation factors involved in secondary metabolite accumulation [28]. When *S. miltiorrhiza* was treated with MeJA, an extensive transcriptional reprogramming of metabolism was triggered, and the biosynthesis of active ingredients was dramatically increased [30]. In addition, it was also shown that the biosynthesis of phenolic acids is stimulated by MeJA treatment [5,9,31,32], and the expression levels of most of the genes involved in the biosynthesis of bioactive compounds are induced by MeJA at different levels [8,9,28,32,33].

In addition, when a plant is subjected to biotic and abiotic stresses, it produces secondary metabolites that function as direct defenses [25], and these metabolites may have important medicinal value. Overexpression of tomato *prosystemin* (*LePS*) in *S. miltiorrhiza* enhanced resistance the pest, while the production of secondary metabolites and the level of endogenous MeJA was increased [34]. Moreover, as described above, overexpressing *JMT* in plants elevated the level of endogenous MeJA, and transgenic plants exhibited constitutive expression of jasmonate-responsive genes [21], while most genes involved in the biosynthesis of Sal B and RA could be induced by jasmonates in *S. miltiorrhiza* [8,9,28,32,33]. Thus, we inferred that, if the level of endogenous MeJA were elevated, a series of defense responses mediated by MeJA may be elicited, a group of jasmonate-responsive genes would be activated, and the accumulation of secondary metabolites could be enhanced.

Thus, we developed a novel strategy to enhance the production of phenolic acids in *S. miltiorrhiza* simultaneously using genetic manipulation and MeJA induction. On the one hand, we overexpressed *SmJMT* in *S. miltiorrhiza* aiming to activate the expression of genes responsible for biosynthesis of phenolic acids and MeJA. On the other hand, by enhancing the level of endogenous MeJA, we intended to elicit a series of biomechanisms to elevate the accumulation of secondary metabolites. After obtaining the overexpressing *SmJMT* plants, transcriptome analysis was carried out on the above transgenic plants and the untransformed control plants. The differentially expressed genes (DEGs) involved in phenolic acids biosynthesis and the α-linolenic acid metabolism pathway were identified. Finally, the contents of RA, Sal B, total phenols, total flavonoids, and endogenous MeJA in transgenic and control lines were analyzed using different biological techniques.

## 2. Results

### 2.1. Isolation and Sequence Analysis of SmJMT

**Figure 1:**
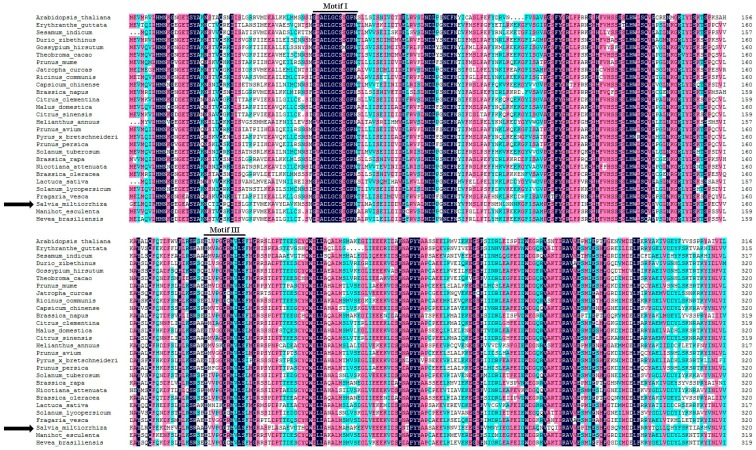
Multiple-sequence alignment of the jasmonic acid (JA) carboxyl methyltransferase (JMT) conserved amino-acid sequences from *Salvia miltiorrhiza* and selected known JMTs from other species, including the binding sites (motifs I and III are indicated) of *S*-adenosyl-l-methionine. The *S. miltiorrhiza* sequences are marked with a black arrow.

Using PCR amplification, the full-length complementary DNA (cDNA) of *SmJMT* was obtained and submitted to GenBank with the accession number MH136806. The cDNA fragment contained a 1167-bp open reading frame (ORF), encoding a predicted 389-amino-acid polypeptide with an isoelectric point of 5.98 and a molecular mass of 43.5 kDa. The amino-acid sequence contains all the characteristic elements of the *S*-adenosyl-l-methionine-dependent methyltransferases [35]; two conserved binding sites of motifs I and III of *S*-adenosyl-l-methionine (SAM) [36,37], the signature of SABATH gene family members [20,21], were found using multiple-sequence alignment (Figure 1).

**Figure 2:**
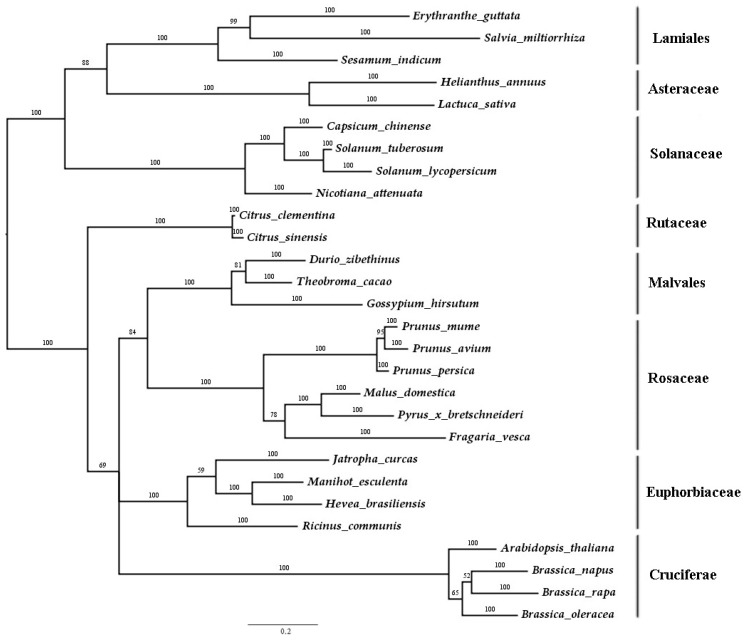
Phylogenetic tree based on JMT from *S. miltiorrhiza* and other species. The tree was constructed using Bayesian inference implemented in MrBayes, based on the amino-acid sequences of *SmJMT* and other species of JMTs under the model of JTT + I + G. The species taxonomy is indicated on the tree.

To determine the evolutionary relationship of the *SmJMT* with the members of JMTs from other species, an unrooted phylogenetic tree was constructed using the amino-acid sequences of *S. miltiorrhiza* JMT and 27 JMTs from other species (Figure 2). Phylogenetic analysis showed that *SmJMT* was most closely related to *SiJMT* (*S. indicum* jasmonic acid carboxyl methyltransferase) and *EgJMT* (*Erythranthe guttata* jasmonic acid carboxyl methyltransferase), both of which belong to the Lamiales order (Figure 2). Furthermore, the species belonging to same family were classed into the same clade (Figure 2), suggesting that the cluster relationship of JMT proteins from different species is consistent with the traditional taxonomy.

### 2.2. Generation of Transgenic S. miltiorrhiza Plants

Transgenic *S. miltiorrhiza* plants overexpressing *SmJMT* were obtained in our laboratory by *Agrobacterium*-mediated transformation. After selective culturing on a glufosinate/ammonium medium, resistant plants were confirmed through PCR amplification to contain an expected 929-bp fragment of the CaMV 35S promoter (Figure S1A). Real-time PCR analyses demonstrated that *SmJMT* was obviously overexpressed at the transcriptional level in lines OEJ-2, OEJ-5, OEJ-7, OEJ-8, OEJ-9, and OEJ-10 (OEJ stands for overexpressed *SmJMT*) (Figure S1B). Since expression was significantly higher in OEJ-7 and OEJ-10 than in the other lines and the non-transformed control, we chose them for further analysis. Due to OEJ-10 being the most highly expressed line, we chose it to do the transcriptome sequencing. However, there was no phenotypic change between transgenic lines and control lines.

### 2.3. Overexpression of SmJMT Enhances Production of Salvianolic and Rosmarinic Acids inTransgenic *S. miltiorrhiza*

**Figure 3:**
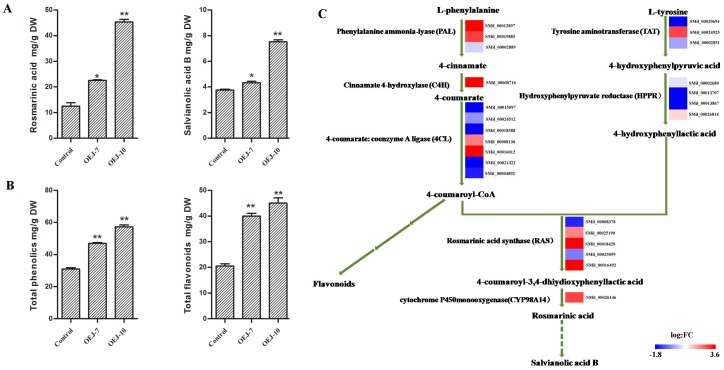
(**A**) Concentrations of rosmarinic acid (RA) and salvianolic acid (Sal B) in root extracts from control and transgenic OEJ-7 and OEJ-10 plants. All data are means of three replicates, with error bars indicating SD. ** Values are significantly different from the control at *p* < 0.01. (**B**) Concentrations of total phenolics and total flavonoids in root extracts from control and transgenic OEJ-7 and OEJ-10 plants. All data are means of three replicates, with error bars indicating SD. * and ** Values are significantly different from the control at *p* < 0.05 and *p* < 0.01, respectively. (**C**) Differentially expressed genes (DEGs) involved in the pathway for salvianolic acid biosynthesis between OEJ-10 and the control. For each gene, relative expression (OEJ-10 versus control) is represented as log_2_FC. The color scale is shown at the bottom. Higher expression levels are shown in red.

To further characterize how the production of phenolic acids was modified in our *SmJMT* overexpression transgenic plants, we extracted the phenolic acids and separated them via LC/MS (Figure S2). The results show that the concentrations of both RA and Sal B, which are the two major hydrophilic active pharmaceutical ingredients in *S. miltiorrhiza*, were increased significantly compared with levels in control samples. We detected the concentrations of RA and Sal B in all the transgenic plants with overexpressed *SmJMT*. The highest concentrations were found in transgenic OEJ-10. Compared with the control, OEJ-10 showed a 3.61-fold increase in RA and a 2.00-fold increase in Sal B (Figure 3A). In OEJ-7, concentrations of RA and Sal B were approximately 1.80- and 1.15-fold higher than those of the control (Figure 3A).

### 2.4. Transgenic Plants Show Higher Levels of Total Phenolics and Total Flavonoids

The results above showed that overexpression of *SmJMT* modified the accumulation of two non-flavonoid phenolic acids, RA and Sal B, while the phenolics and flavonoids share an upstream core phenylpropanoid metabolism with Sal B [38]. To determine whether the upregulation of RA and Sal B could cause activation of the phenylpropanoid pathway and provide substrates for the biosynthesis of other types of end product, global assays for phenolics and flavonoids of transgenic plants and control plants were performed. The results showed that total phenolics and total flavonoids accumulated at higher levels in the roots of the overexpression line than in the control sample. The highest concentrations of total phenolics and total flavonoids were also found in transgenic OEJ-10 (Figure 3B). Differences were significant and corresponded to a 1.85-fold increase in the total phenolics content of OEJ-10 roots and a 2.20-fold increase in the total flavonoid content. In OEJ-7, concentrations of total phenolics and total flavonoids were approximately 1.56- and 1.94-fold higher than those of the control (Figure 3B).

### 2.5. Transcriptomic Analysis of S. miltiorrhiza SmJMT Overexpression and Control Plants

**Figure 4:**
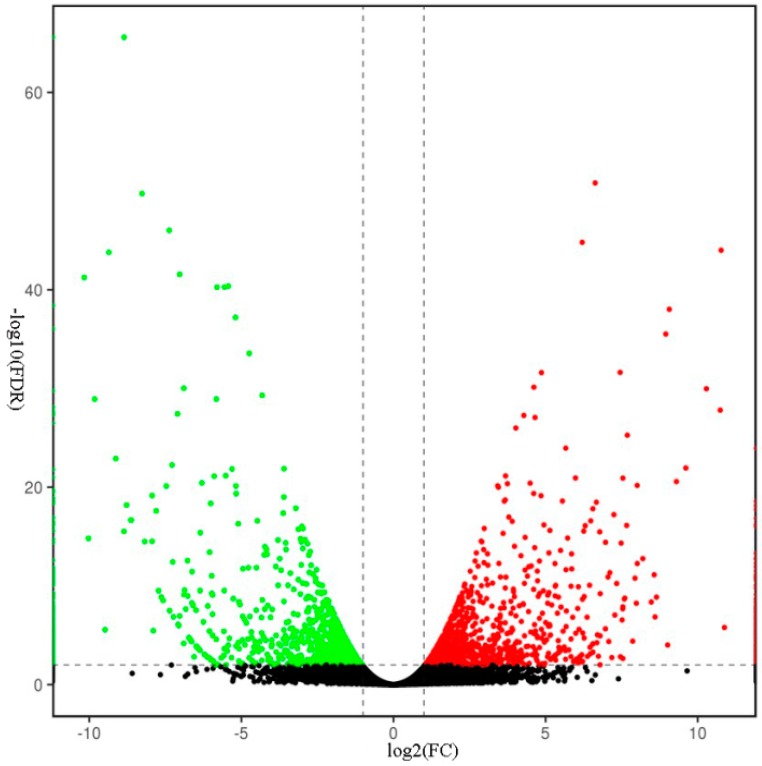
Volcano plot of DEGs between OEJ-10 and the control. Red points represent the DEGs that were upregulated. Black points represent the DEGs without statistically significant differences. Green points represent the DEGs that were downregulated.

In order to detect the differentially expressed genes between *SmJMT* overexpression transgenic and control plants, and the genes regulated by *SmJMT*, RNA sequencing (RNA-seq) experiments were performed, and the global expression profiles of OEJ-10 and the control were compared. Through Illumina deep sequencing, approximately 26.03 and 26.88 million high-quality clean reads were obtained from OEJ-10 and the control, respectively. The average length of each read was 296 bp. The Q30 values (percentage of sequences with a sequencing error rate <0.1%) for OEJ-10 and the control were 93.45% and 93.38%, respectively. Principal component analysis (PCA) showed that the two groups of samples were distributed in different regions of the spaces, and were distinguished clearly. It indicated that there are differences between the two groups of samples (Figure S3). The control samples were relatively concentrated, indicating that the biological homogeneity of the control samples was better (Figure S3). According to the criteria of differential gene expression screening, there were 2052 genes showing significant differences in expression between the OEJ-10 and control plants, with 998 genes being upregulated and 1054 genes downregulated in OEJ-10 when compared with expression in control (Figure 4 and Table S1).

**Figure 5:**
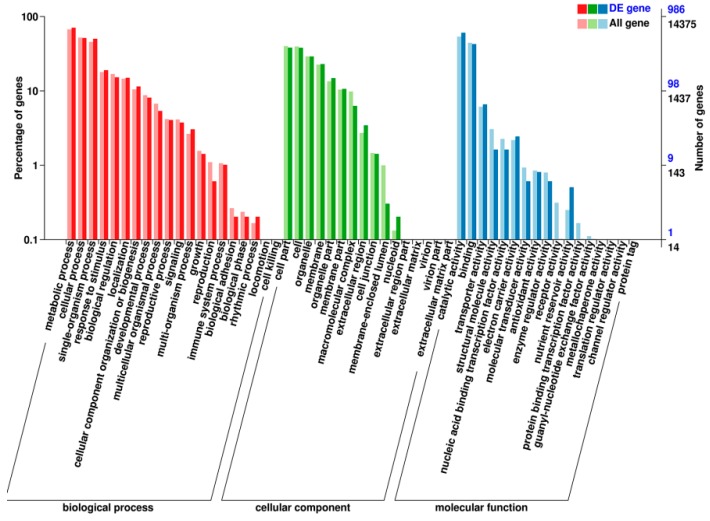
The second Gene Ontology (GO) classification annotation of DEGs between OEJ-10 and the control. The *X*-axis represents GO classification; red represents a biological process, green represents a cellular component, and blue represents a molecular function. The left *Y*-axis represents the percentage of DEGs with respect to all genes. The right *Y*-axis represents the number of genes.

The Gene Ontology (GO) analysis showed that a total of 14,375 unigenes were annotated in this manner, including 986 DEGs (Figure 5 and Table S2). The GO terms of three categories, namely biological process, cellular component, and molecular function, were assigned to categorize the function of the predicted unique sequences of *S. miltiorrhiza*. In many cases, multiple terms were assigned to the same transcript [39]. This categorization resulted in 1628 DEGs assigned to cellular component, 2573 DEGs to biological process, and 1163 DEGs to molecular function. The GO terms of “binding” (GO: 0005488) and “catalytic activity” (GO: 0003824) of molecular function; “cell part” (GO: 0044464) and “cell” (GO: 0005623) of cellular component; and “cellular process” (GO: 0009987) and “metabolic process” (GO: 0008152) of biological process were predominantly represented (Figure 5 and Table S2). Furthermore, the enriched GO terms “l-phenylalanine metabolic process” (GO: 0009694), “jasmonic acid metabolic process” (GO: 0006558), and “response to extracellular stimulus” (GO: 0009991) correlate well with the biosynthetic pathways for phenolic acids, α-linolenic acid metabolism, and plant defense.

**Figure 6:**
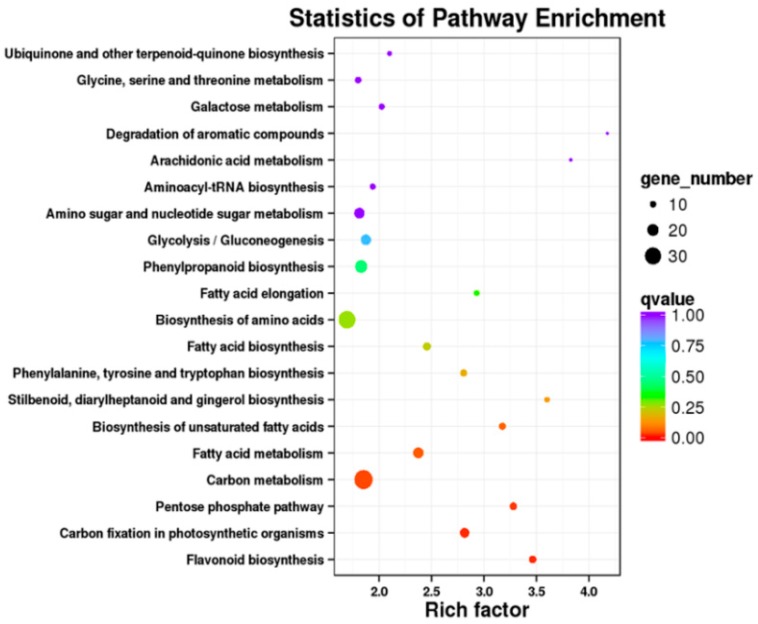
Kyoto Encyclopedia of Genes and Genomes (KEGG) pathway enrichment analysis of DEGs between OEJ-10 and the control. The *X*-axis represents enrichment factor; the *Y*-axis represents the pathway. The circles represent the KEGG pathways. The color of the circle represents the *q*-value. Lower *q*-values are shown in red.

In addition, the Kyoto Encyclopedia of Genes and Genomes (KEGG) analysis showed that a total of 379 DEGs could be to assigned to KEGG pathways, with enrichment in pathways including phenylalanine, tyrosine, and tryptophan biosynthesis (ko00400) and phenylpropanoid biosynthesis (ko00940), which are connected with the biosynthetic pathways for phenolic acids, phenylalanine, and tyrosine (Figure 6 and Table S3). Both GO terms and KEGG pathways of transcriptome analysis were correlated with the phenylalanine metabolic process, indicating that *SmJMT* could be correlated with the biosynthetic pathways for phenolic acids in *S. miltiorrhiza*.

### 2.6. DEGs Involved in α-Linolenic Acid Metabolism and Determination of Endogenous MeJA Levels

**Figure 7:**
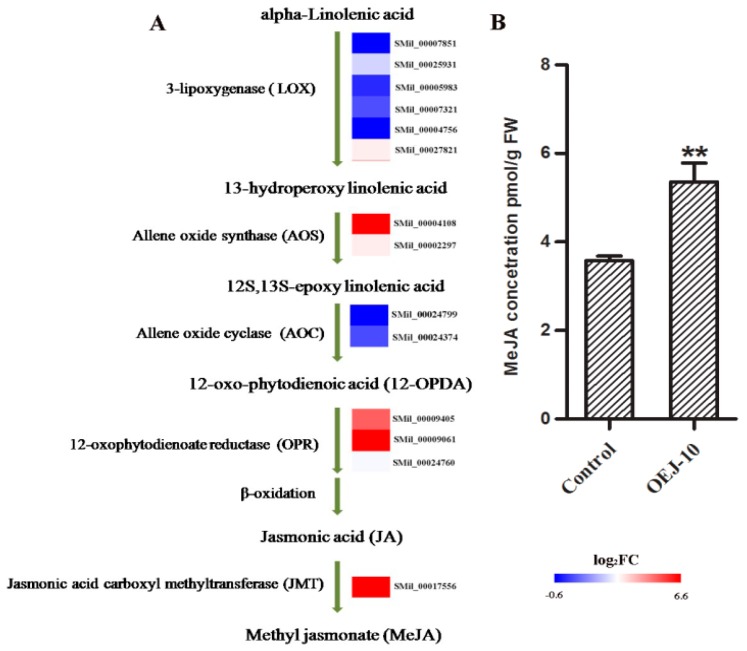
(**A**) DEGs involved in the pathway for MeJA biosynthesis between OEJ-10 and the control. For each gene, relative expression (OEJ-10 versus control) is represented as log_2_FC. The color scale is shown at the bottom. Higher expression levels are shown in red. (**B**) Concentrations of MeJA in leaf extracts from transgenic line OEJ-10 and the control. All data are means of three replicates, with error bars indicating SD. ** Values are significantly different from the control at *p* < 0.01.

To determine whether overexpressing *SmJMT* affected the expression of genes closely associated with the pathway of α-linolenic acid metabolism, which finally leads to MeJA biosynthesis, we investigated the changes in expression of those genes. These genes include *SmLOX*, *SmAOS*, *SmAOC*, *SmOPR*, and *SmJMT*. The KEGG analysis revealed that a total of 14 genes assigned to the α-linolenic acid metabolism pathway (ko00592) and three DEGs relevant to MeJA biosynthesis were found (Figure 7A). Among the DEGs encoding putative *SmAOS*, *SmOPR*, and *SmJMT*, all of them have one unigene transcriptionally activated and being upregulated (Figure 7A and Table S4). For further detecting the endogenous MeJA levels of transgenic plants, the concentrations of endogenous MeJA in fresh leaves from OEJ-10 and control plants were determined using ELISA. According to the optical density (OD) values of samples, the concentrations of MeJA were 3.57 ± 0.08 pmol∙g^−1^ for the control and 5.36 ± 0.30 pmol∙g^−1^ for OEJ-10, respectively. MeJA concentrations were significantly higher for the transgenic OEJ lines (Figure 7B).

### 2.7. DEGs Involved in the Pathway for Salvianolic Acid Biosynthesis

To evaluate whether upregulated expression of *SmJMT* could modify the activation of the key enzymes in the pathway for salvianolic acid biosynthesis, all of the putative enzyme genes in this pathway were examined through transcriptome analysis. A total of 25 unique sequences that encode seven enzymes involved in the biosynthetic pathway of salvianolic acid were present in the RNA-seq dataset, including 12 DEGs (Table S5). These enzymes were SmPAL, SmC4H, Sm4CL, SmTAT, SmHPPR, SmRAS, and SmCYP98A14 (Figure 3C). According to the transcriptome data, among the 12 DEGs in the salvianolic acid biosynthesis pathway, nine were upregulated in the OEJ-10 plants, while three were downregulated (Figure 3C and Table S5). Following comprehensive analysis of the values of counts and the annotation of the RNA-seq dataset, the expression of *SmPAL_1_* (*SMil_00019885* Accession No: KF462460), *SmC4H* (*SMil_00000716* Accession No: DQ355979), *Sm4CL_3_* (*SMil_00016012* Accession No: KF220556), *SmTAT* (*SMil_00024925* Accession No: DQ334606), *SmRAS* (*SMil_00025190* Accession No: FJ906696), and *SmCYP98A14* (*SMil_00026146* Accession No: HQ316179) demonstrated significant upregulation in OEJ-10, while the expression of *SmHPPR* (*SMil_00002680* Accession No: DQ09974) was not significantly upregulated.

### 2.8. Confirmation of RNA-Seq Data by qRT-PCR Analysis

**Figure 8:**
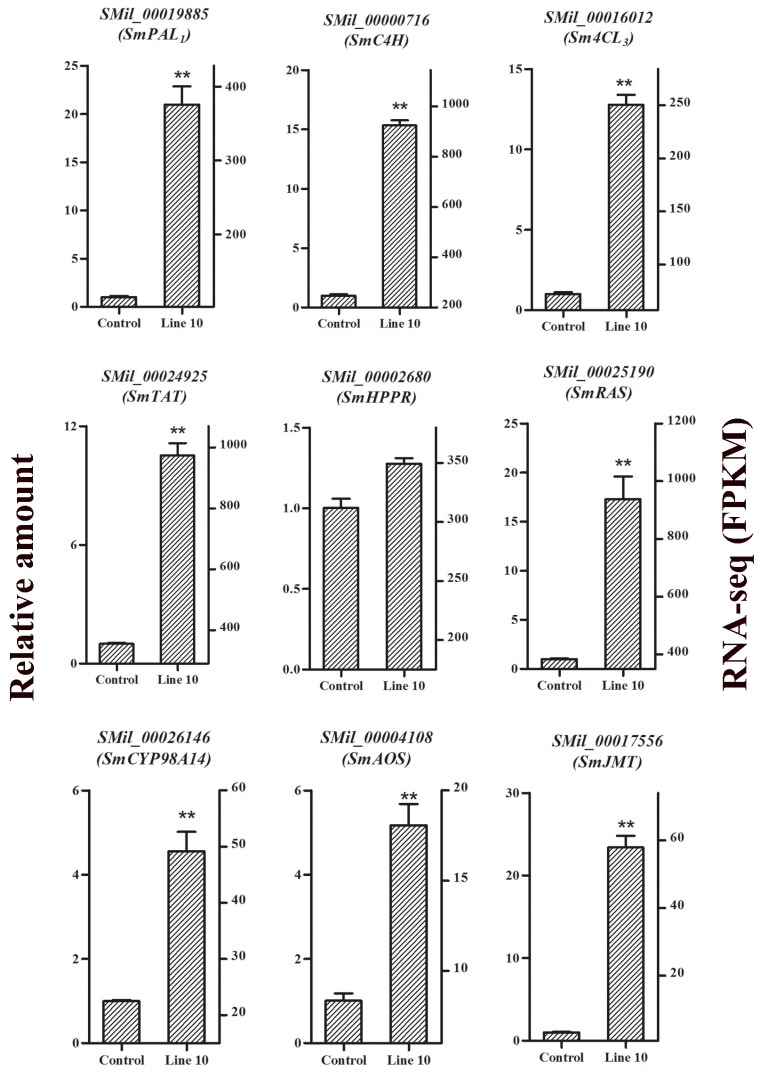
Validation by qRT-PCR of nine genes involved in the salvianolic acid biosynthesis and MeJA biosynthesis pathways in the control and transgenic line OEJ-10. All data are means of three replicates, with error bars indicating SD. ** Values are significantly different from the control at *p* < 0.01.

To validate the RNA-seq data for differential gene expression between the control and transgenic OEJ lines, the expression levels of genes encoding nine key enzymes involved in the salvianolic acid biosynthesis and α-linolenic acid metabolism were analyzed by qRT-PCR (Figure 8). According to the statistical analysis of qRT-PCR, *SmPAL_1_*, *SmC4H*, *Sm4CL_3_*, *SmTAT*, *SmHPPR*, *SmRAS*, *SmCYP98A*, *SmAOS*, and *SmJMT* were upregulated in transgenic lines compared with the control (Figure 8). On the other hand, based on the screening conditions of DEGs, the expression of *SMil_00019885*, *SMil_00000716, SMil_00016012*, *SMil_00024925*, *SMil_00025190*, *SMil_00026146*, *SMil_00002680*, *SMil_00004108*, and *SMil_00017556* was upregulated in transgenic lines compared with the control (Tables S4 and S5). Thus, through statistical analysis, the relative expression levels of these genes were shown to be consistent with those of the RNA-seq data, which indicated the accuracy of the results of the latter (Figure 8).

## 3. Discussion

Among medicinal plants, *S. miltiorrhiza* is an ideal and representative model for studying transcriptional regulation, and phenolic acid biosynthesis became a new research focus [7]. Fortunately, the *S. miltiorrhiza* genomic sequence was published [4], and this provides a good opportunity for studying the function of many valuable genes in *S. miltiorrhiza*. In this article, we report on the isolation, bioinformatics analysis, molecular characterization, and preliminary function analysis of the *JMT* gene from *S. miltiorrhiza* encoding jasmonic acid carboxyl methyltransferse. Phylogenetic analysis of the SABATH gene family of *S. miltiorrhiza* and *Arabidopsis* suggests that *SmJMT* and *AtJMT* are apparent orthologs and may have the same function [20]. Overexpressing JMT in plants can elevate the level of endogenous MeJA and improve resistance against external environmental stimuli [21,24,26]. Thus, we sought to detect the effect of JMT on the biosynthesis of MeJA in *S. miltiorrhiza* and study the function of JMT in regulating the expression of genes involved in the phenolic acid biosynthesis pathway, as well as the impact on accumulation of secondary metabolites in *S. miltiorrhiza*.

After obtaining the full-length cDNA of *SmJMT*, we constructed a *SmJMT* overexpression vector and transferred it into *S. miltiorrhiza*. Then, after obtaining *SmJMT* overexpression in transgenic *S. miltiorrhiza* lines, we compared the MeJA levels between control and transgenic lines. The results showed that overexpression of *SmJMT* significantly changed the level of endogenous MeJA in *S. miltiorrhiza* (Figure 7B). In addition, transcriptome analysis showed that the three DEGs (*AOS*, *OPR*, and *JMT*) in α-linolenic acid metabolism were upregulated in transgenic lines, while no genes were downregulated, suggesting that the α-linolenic acid metabolism pathway was obviously activated in transgenic line OEJ-10 (Figure 7A). Furthermore, AOS is the major control point of MeJA biosynthesis and the first specific enzyme [17,40,41]. The transcription level of *SmAOS* was significantly upregulated in OEJ-10 according to the transcriptome data. This strong induction of *SmAOS* may contribute to the biosynthesis of MeJA, which was consistent with the results of the ELISA experiment (Figure 7).

Jasmonic acid carboxyl methyltrans-ferase (JMT) is a key enzyme for jasmonate-regulated plant responses and defense response [17,21,24,42]. Based on previous studies, the expression of primary enzymes involved in the phenylpropanoid and tyrosine-derived pathways are upregulated by jasmonates, which could enhance the biosynthesis of phenolic acids [8,9,28,32,33]. Furthermore, the elicitation of defense responses could lead to the accumulation of secondary metabolites [25,34]. Therefore, we designed a strategy to elevate the content of phenolic acids through overexpression of *JMT* in *S. miltiorrhiza*. According to the transcriptome data, genes involved in the phenylpropanoid pathway (*SmPAL*, *SmC4H*, and *Sm4CL*) and in the tyrosine-derived pathway (*SmTAT* and *SmHPPR*), as well as *SmRAS* and *SmCYP98A*, were upregulated in transgenic lines compared with the control (Figure 3C). Meanwhile, the contents of RA, Sal B, total phenolics, and total flavonoids in transgenic lines were significantly elevated over those of the control (Figure 3A,B). The results indicated that overexpression of *SmJMT* significantly increased the contents of phenolic acids by activating the phenylpropanoid and tyrosine-derived pathways. *SmJMT* may play an important role in the regular expression of functional genes contributing to the accumulation of phenolic acids. Our results demonstrate that production of salvianolic acid could be improved by overexpression of *SmJMT* in *S. miltiorrhiza* using genetic engineering techniques. Therefore, our research on *SmJMT* overexpression in *S. miltiorrhiza* provides a good foundation for further study of the function of JMT in plants.

MeJA treatment is the most commonly used method of eliciting herbivore resistance in many different plant species, and a series of JA-mediated defense responses are quickly elicited when plants are exposed to volatile MeJA [43,44,45]. In brief, when treated with exogenously applied MeJA, plants could elicit most defense-resistant responses by JA [25]. However, following overexpression of the plastidic flax *AOS* cDNA in transgenic potato plants, even though the plants exhibited six- to 12-fold increased levels of JA, this increase did not activate jasmonate-responsive genes [46]. The reason for this may be that the free acid JA could not move across the cellular membrane without a carrier [21,47], while MeJA, which diffuses through the membranes, may act as an intracellular regulator and a diffusible intercellular signal transducer mediating intra- and interplant communications [21]. MeJA also plays its own role in developmental processes and defense responses. Therefore, the effects of treatment with exogenously applied MeJA may be different from those of enhanced endogenous MeJA levels in plants. Improvement of the defense ability of pharmaceutical crops through spraying of jasmonates is not feasible, while strengthening the stress resistance of medicinal herbs by elevating endogenous MeJA levels via genetic engineering would represent a new strategy. Furthermore, the plant defense is related to plant secondary metabolism. The improvement of plant defense could promote the accumulation of secondary metabolites. The defense mechanisms, resistance effects, and plant responses need to be further studied.

## 4. Materials and Methods

### 4.1. Isolation of the SmJMT Gene

Plant materials of leaves from *S. miltiorrhiza* were collected following a method described previously [20]. Total RNA was extracted from the *S. miltiorrhiza* leaf tissue with a Plant RNA Kit (OMEGA, Houston, TX, USA). RNA quantity was determined using a NanoDrop 2000C Spectrophotometer (Thermo Scientific, Wilmington, DE, USA). First-strand cDNA was synthesized using a Prime-Script RT Master Mix (TaKaRa, Beijing, China) according to the manufacturer’s protocol. The full-length cDNA coding sequence for *SmJMT* was amplified from leaf cDNA with gene-specific primers *SmJMT*-F and *SmJMT*-R (Table S6), both of which were designed according to the phylogenetic analysis of the *SmSABATH* gene family [20] and the unigene sequence (*SMil_00017556*) available in the *S. miltiorrhiza* genomic database [4]. Amplification was achieved using PrimeSTAR^®^ HS DNA Polymerase (TaKaRa, Beijing, China), and the PCR reaction was performed on a FlexCycler thermocycler (Analytikjena, Jena, Germany) in a 50-μL final volume comprising 2.5 U/μL PrimeSTAR^®^ HS DNA Polymerase, 100 ng of first-strand cDNA, 500 nM each primer, 10 μL of 5× PrimeSTAR Buffer, and 2.5 mM deoxynucleoside triphosphate (dNTP) mixture, under the following conditions: cDNA was denatured at 94 °C for 3 min followed by 35 cycles of amplification (94 °C for 30 s, 51 °C for 30 s and 72 °C for 72 s), and then extension at 72 °C for 10 min. The PCR fragments were purified using a DNA Gel Extraction Kit (Tiangen Beijing, China), inserted into pMD19T-vector (Takara, Beijing, China), and then sequenced by Beijing Genomics Institute (Shenzhen, Guangdong, China).

### 4.2. Multiple-Sequence Alignment and Phylogenetic Analysis

Multiple-sequence alignment of the JMT conserved amino-acid sequences from *S. miltiorrhiza* and selected known JMTs from other species (Table S7) was performed with the DNAMAN program (Lynnon Corporation, San Ramon, CA, USA). Conserved blocks were obtained with the online program Gblocks 0.91b (http://www.phylogeny.fr/one_task.cgi?task_type=gblocks) [48]. Phylogenetic trees were constructed using Bayesian inference implemented in MrBayes [49,50] with the amino-acid sequences of the *SmJMT* and homolog from other species under the model of JTT + I + G. The model was chosen using the program ProtTest [51]. The phylogenetic tree was represented with the help of Treeview1.61 software [52].

### 4.3. Vector Construction and Transformation

In order to construct *SmJMT* overexpression vectors, the pMD19T-*JMT* plasmid was used as a template to amplify *SmJMT* with the primers pDONR207-*SmJMT*-F/pDONR207-*SmJMT*-R (Table S6), which contained *att*B1/*att*B2 sites. PCR amplification followed the description above. The PCR products were purified and cloned into entry vector pDONR207, using the BP recombination reaction, and then transferred into the destination vector pEarleyGate202 with the LR reaction according to the protocol from the Gateway Technology manufacturer (Invitrogen, Carlsbad, CA, United States) (Figure S4). The pDONR207-*SmJMT* and pEarleyGate202-*SmJMT* plasmid were sequenced by Beijing Genomics Institute (Shenzhen, Guangdong, China). Finally, the pEarleyGate202-*SmJMT* plasmid was transferred into *Agrobacterium tumefaciens* EHA105 using the freeze–thaw method [53].

An *Agrobacterium*-mediated gene transfer method was performed to generate transgenic plants with leaf explants from sterile *S. miltiorrhiza* cultured on Murashige and Skoog (MS) basal medium under the conditions described previously [54,55]. After transformation, MS with 1 mg∙L^−1^ naphthalene acetic acid, 10 mg∙L^−1^ 6-benzyl-aminopurine, 10 mg∙L^−1^ glufosinate/ammonium, and 200 mg∙L^−1^ cefotaxime as the selection medium was used to culture the transgenic explants. They were transferred to fresh selection medium at one-week intervals. Developing shoots were excised and placed on ½ MS selection medium supplemented with 10 mg∙L^−1^ glufosinate/ammonium, and 200 mg∙L^−1^ cefotaxime for root induction [34]. After two weeks, the rooted transgenic plants were propagated through several generations to expand the culture on the MS basic medium.

### 4.4. PCR Detection and qRT-PCR Analysis

Genomic DNA was obtained from leaves of one-month-old transgenic and control plants using the CTAB Plant Genomic DNA Rapid Extraction Kit (Aidlab, Beijing, China) according to the manufacturer’s protocol. A pair of gene-specific primers (pEarleyGate202-35S-F/R) (Table S6) was used to amplify a 929-bp fragment from genomic DNA of transgenic and control plants. PCR reactions were also performed on a FlexCycler thermocycler (Analytikjena, Jena, Germany) with a 20-μL final volume comprising 10 μL of 2× Taq PCR master Mix (Tiangen, Beijing, China), 500 nM each primer, and 100 ng of template DNA. All PCR reactions were carried out as follows: preheating at 94 °C, then 35 cycles of amplification at 94 °C for 30 s, 58 °C for 30 s, and 72 °C for 1 min, followed by a final elongation of 10 min at 72 °C. The positive control was the pEarleyGate202-*SmJMT*vector, while genomic DNA from wild-type plants served as the negative control. Amplified products were electrophoresed on a 1.0% agarose gel.

Total RNA extraction, RNA quantity determination, and first-strand cDNA synthesis from transgenic and control plants followed the method described above. Quantitative PCR was carried out on a Light Cycler 96 Instrument (Roche, Basel, Switzerland) in a 20-μL final volume comprising 10 μL of SYBR Premix Ex Taq II (Takara, Beijing, China), 20 ng of first-strand cDNA, and 500 nM each primer. The reactions were performed in triplicate under the following conditions: initial thermal cycling at 95 °C for 30 s, followed by 45 cycles of 95 °C for 10 s and 60 °C for 30 s. *Smβ-actin* (DQ243702) was selected as a reference gene [20,56]. Relative expression was calculated by the 2^−ΔΔ*C*t^ method [57], and the relative expression levels were analyzed as means ± standard deviation (SD) of the biological triplicates. The lengths of the amplicons were between 100 and 250 bp. Quantitative primers are listed in Table S6.

### 4.5. Extraction of MeJA and Determination of Its Concentration

Fresh leaves from transgenic plants lines and control plants were used to investigate the MeJA concentration levels. Tissues (100 mg) were ground in liquid nitrogen, and 9 mL of phosphate-buffered saline (PBS; pH 7.4) was added. The extracts were then centrifuged at 8000× *g* at 4 °C for 30 min, and the upper layer was collected. Based on the method of detecting the endogenous jasmonic acid (JA) in *S. miltiorrhiza* [38], the endogenous MeJA of both transgenic and control plants was measured with a Plant MeJA ELISA Kit (mlbio, Shanghai, China) according to the manufacturer’s protocol. A standard curve of optical density (OD) versus MeJA concentration at 0, 125, 250, 500, 1000, and 2000 pmol∙L^−1^ was produced by testing a set of calibration standards. By comparing OD values with the standard curve, the content of MeJA in each sample was determined. The intensity of the final reaction color was measured spectrophotometrically at 450 nm to calculate the final MeJA concentration [38].

### 4.6. Extraction of Total Phenolics and Total Flavonoids and Determination of Their Concentrations

The roots from two-month-old transgenic and control *S. miltiorrhiza* plantlets were air-dried at 20 ± 2 °C. The dried root sample (20 mg) was ground into a powder, and mixed with 1 mL of methanol and acetone (7:3, *v*/*v*) in an ultrasonic bath for 1 h. The mixture was centrifuged at 6000× *g* for 3 min and the supernatant was collected.

The total phenolics content was measured using a modified colorimetric Folin–Ciocalteu method [58] with gallic acid as the standard. The extract solution (100 μL) was mixed in a centrifuge tube with 500 μL of the Folin–Ciocalteu reagent in darkness for 8 min, then incubated with 400 μL of sodium carbonate solution (7.5% *w*/*v*) at 40 °C for 30 min. Absorbance was measured at 765 nm against a reagent blank without the extract, and measurements were carried out in triplicate. The calibration equation for gallic acid was *y* = 0.0073*x* + 0.1023 (*R*^2^ = 0.9995).

Total flavonoid content was determined following the procedure of Dewanto et al. [59] with epicatechin as the standard. The test sample (100 μL) was placed in a centrifuge tube, and 800 μL of 60% ethanol was added followed by 20 μL of 5% NaNO_2_. After 6 min, 20 μL of 10% AlCl_3_ was added. After another 6 min, 60 μL of 4% NaOH was added, and then the solution was mixed and the absorbance was measured at 510 nm against a reagent blank without the extract; measurements were carried out in triplicate. The calibration equation for epicatechin was *y* = 0.0022*x* + 0.0737 (*R*^2^ = 0.9900).

### 4.7. LC/MS Analysis of Phenolic Compounds

Roots harvested from transgenic *S. miltiorrhiza* transplanted for two months and control plantlets were air-dried at 20 ± 2°C. Dried roots were then ground to a fine powder in a mechanical grinder with a 2-mm-diameter mesh. Samples (30 mg) were extracted with 500 μL of 75% methanol in an ultrasonic bath for 20 min, and then centrifuged at 12000× *g* for 6 min. The residual pellet was re-extracted twice, and all supernatants were combined. Finally, the extracted samples were filtered through a 0.2-μm Millipore filter and analyzed by LC/MS.

For LC/MS, extracts were applied to an Agilent 1260 HPLC system coupled to an Agilent 6460 QQQ LC–MS system (Agilent Technologies, Palo Alto, CA, USA), an HPLC system equipped with a pump (Agilent G1312B), an auto-sampler (Agilent G7127A), and a column temperature controller (Agilent G1316A). Chromatography separation was performed with a Welch UItimate XB-C_18_ column (150 × 2.1 mm, 3 μm particle size) at a flow rate of 0.4 mL·min^−1^ (temperature 30 °C) and 5 μL of sample was injected. The mobile phase comprised Solvent A (acetonitrile) and Solvent B (0.1% formic acid in deionized water), and followed a solvent gradient profile: 0–6 min, A 20–60% and B 80–50%; 6–7 min, A 60–20% and B 50–80%; 7–10 min, A 20% and B 80%. The retention times were 3.8 min for RA and 4.1 min for Sal B.

Mass spectra were acquired by a QQQ-MS instrument with an Agilent Jet Stream (AJS) electrospray ionization (ESI) source. Multiple reaction monitoring (MRM) mode was used for the quantification. For phenolic acids, the ionization mode was negative and the selected transitions of *m*/*z* were 359.1→161.1 for RA and 717.2→519.2 for Sal B. The fragmentor voltage was 130 V and the collision energy was 18 eV. The drying gas flow was 10 L∙min^−1^, and the nebulizer pressure was set to 45 psi at a capillary temperature and voltage of 350 °C and 3500 V, respectively. The sheath gas flow was 11 L∙min^−1^ at a temperature of 350 °C.

### 4.8. Transcriptome Analysis and Identification of Differentially Expressed Genes (DEGs)

Two-month-old transgenic and control *S. miltiorrhiza* plants were collected from three biological replicates. Total RNA extraction and RNA quantity determination followed the method described above. The cDNA library preparation and construction were performed as described previously [60]. The libraries were sequenced by Biomarker Technologies Co., Ltd. (Beijing, China) with an Illumina HiSeq4000 platform. The raw transcriptome data were submitted to the National Center for Biotechnology Information (NCBI) with the accession number of SRP155681.

After sequencing of the transcriptome, clean paired-end reads were mapped to the reference genome of *S. miltiorrhiza* [4] using TopHat v2.0.12 [61]. The *S. miltiorrhiza* genome and gene model annotation files were downloaded from genome websites directly (http://www.ndctcm.org/shujukujieshao/2015-04-23/27.html) [4]. Principal component analysis (PCA) was employed to investigate the correlation of biological duplication [62]. Analysis of differential gene expression was performed by Cufflinks [63], and fragments per kilobase of transcript per million mapped reads (FPKM) was used to normalize gene expression levels [64]. The DESeq R package was used to analyze the differential expression of the transgenic and control plants [65]. The false discovery rate (FDR) was controlled by *p*-values, which were corrected by the Benjamini–Hochberg procedure. Genes with |log_2_fold-change| ≥ 2 and an adjusted *p*-value < 0.01, as found by DESeq were considered differentially expressed. Gene Ontology (GO) enrichment analysis of DEGs was performed using the topGO R package [66]. GO terms with corrected *p*-values < 0.01 were considered significantly enriched in the DEGs. To identify significantly over-represented metabolic pathways or signal transduction pathways, all DEGs were mapped to terms in the KEGG (Kyoto Encyclopedia of Genes and Genomes) database [67], and the pathway enrichment analysis was conducted using KOBAS [68].

### 4.9. Verification of RNA-Seq Data by qRT-PCR

To validate the RNA-seq data, seven unigenes involved in salvianolic acid biosynthesis (*SMil_00019885*, *SMil_00000716*, *SMil_00016012*, *SMil_00024925*, *SMil_00002680*, *SMil_00025190*, and *SMil_00026146*) and two unigenes involved in α-linolenic acid metabolism (*SMil_00004108* and *SMil_00017556*) were selected for expression analysis through qRT-PCR experiments. Transcriptome data showed that mostly those genes had significant changes in expression between control and transgenic OEJ lines. The qRT-PCR experiment implementation and statistical analysis followed the description above. Quantitative primers are listed in Table S6.

## Supplementary Materials

Supplementary materials can be found at http://www.mdpi.com/1422-0067/19/12/3788/s1. Table S1: Overview of up- and down-regulated genes; Table S2: Results of topGO enrichment analysis of DEGs; Table S3: Overview of the significant enrichment of the KEGG pathways; Table S4: DEGs involved in the α-Linolenic acid metabolism; Table S5: DEGs involved in the salvianolic acid biosynthesis; Table S6: Primer pairs used in the paper; Table S7: List of the JMT from different species; Figure S1: Expression of *SmJMT* in control and overexpression transgenic lines; Figure S2: Principal Component Analysis (PCA) about the correlation of biological duplication of the transcriptome sequencing samples; Figure S3: Mass chromatograms of standards (RA and Sal B) and samples (Control, OEJ-7 and OEJ-10); Figure S4: The *SmJMT*-overexpression vectors construction process with Gateway technology.
