# Supplementary material for: Molecular Characterization and Overexpression of SmJMT Increases the Production of Phenolic Acids in Salvia miltiorrhiza
Source: Int J Mol Sci. 2018 Nov 28;19(12):3788. doi: 10.3390/ijms19123788 (PMC6321555; doi:10.3390/ijms19123788)
Supplement: Supplementary file 1 [file ijms-19-03788-s001.zip › Supplementary caption.docx]

**Supplementary caption**

**Supplementary Figure 1**. Expression of *SmJMT* in control and overexpression transgenic lines.

**(A)** PCR detection. M, DL2000 DNA marker; band sizes (bp) are shown on left side. Lane 0, negative control; Lane 1, positive control; Lanes 2–21, different transgenic lines.

**(B)** Real-time quantitative PCR analysis; all data are means of three replicates, with error bars indicating SD; * and ** values are significantly different from control at *P* < 0.05 and *P* < 0.01, respectively.

**Supplementary Figure 2**. Principal Component Analysis (PCA) about the correlation of biological duplication of the transcriptome sequencing samples. Red points represent the control samples (T0). The blue points represent the overexpression samples (T10). The X, Y and Z axes represent the first, second and third principal components respectively, and the contribution degree of each principal component is shown in brackets.

**Supplementary Figure 3.** Mass chromatograms of standards (RA and Sal B ) and samples (Control, OEJ-7 and OEJ-10).

**Supplementary** **Figure 4.** The *SmJMT*-overexpression vectors construction process with Gateway technology.

**Supplementary Table 1**. Overview of up- and down-regulated genes.

**Supplementary Table 2**. Results of topGO enrichment analysis of DEGs.

**Supplementary Table 3**. Overview of the significant enrichment of the KEGG pathways.

**Supplementary Table 4**. DEGs involved in the α-Linolenic acid metabolism.

**Supplementary Table 5**. DEGs involved in the salvianolic acid biosynthesis.

**Supplementary Table 6**. Primer pairs used in the paper.

**Supplementary Table 7**. List of the JMT from different species.

:

© 2018 by the authors. Submitted for possible open access publication under the terms and conditions of the Creative Commons Attribution (CC BY) license (http://creativecommons.org/licenses/by/4.0/).
