# Supplementary material for: Molecular Characterization and Overexpression of SmJMT Increases the Production of Phenolic Acids in Salvia miltiorrhiza
Source: Int J Mol Sci. 2018 Nov 28;19(12):3788. doi: 10.3390/ijms19123788 (PMC6321555; doi:10.3390/ijms19123788)
Supplement: Supplementary file 1 [file ijms-19-03788-s001.zip › Supplementary Table 4.docx]

**Supplementary Table 4**. DEGs involved in the α-linolenic acid metabolism

| **Gene name** | **RPKM_T0** | **Count_T0** | **RPKM_T10** | **Count_T10** | **DESeq_FDR** | **DESeq_log2FC** | **DESeq_(FDR_0.01_FC_2)_regulated** |
| --- | --- | --- | --- | --- | --- | --- | --- |
| **α-linolenic acid metabolism** | | | | | | | |
| ***3-lipoxygenase ( LOX)*** | | | | | | | |
| *SMil_00007851* | 35.51988 | 1832 | 22.17553 | 1191 | 0.623377664 | -0.618167123 | normal |
| *SMil_00025931* | 4.12911 | 197 | 7.513875 | 354 | 0.601154585 | 0.847086781 | normal |
| *SMil_00005983* | 54.80468 | 2382 | 71.68341 | 2701 | 0.914148344 | 0.184031785 | normal |
| *SMil_00007321* | 8.605224 | 464 | 10.54766 | 575 | 0.615828248 | 0.319656431 | normal |
| *SMil_00004756* | 129.169 | 894 | 101.9495 | 719 | 0.82748569 | -0.300956083 | normal |
| *SMil_00027821* | 153.3561 | 4943 | 314.8841 | 10190 | 0.165655087 | 1.048780972 | normal |
| ***allene oxidesynthase (AOS)*** | | | | | | | |
| *SMil_00004108* | 6.409725 | 135 | 20.5412 | 415 | 0.000357749 | 1.625904485 | up |
| *SMil_00002297* | 9.03323 | 213 | 19.42305 | 439 | 0.026079536 | 1.051854432 | normal |
| ***allene oxide cyclase (AOC)*** | | | | | | | |
| *SMil_00024799* | 123.9752 | 1179 | 106.0002 | 981 | 0.839271272 | -0.256326435 | normal |
| *SMil_00024374* | 100.2456 | 998 | 128.9088 | 1222 | 0.869914157 | 0.30507669 | normal |
| ***12-******oxophytodienoate reductase (OPR)*** | | | | | | | |
| *SMil_00009405* | 68.54803 | 1456 | 152.5363 | 3430 | 0.121766752 | 1.241605428 | normal |
| *SMil_00009061* | 37.7571 | 881 | 94.37575 | 2268 | 0.00032372 | 1.369900387 | up |
| *SMil_00024760* | 51.03274 | 1280 | 100.7987 | 2547 | 0.016131245 | 0.998909838 | normal |
| ***Jasmonic acid carboxyl methyltransferase (JMT)*** | | | | | | | |
| *SMil_00017556* | 0.794101 | 18 | 73.79509 | 1725 | 1.50E-51 | 6.629856114 | up |
