# Supplementary material for: Molecular Characterization and Overexpression of SmJMT Increases the Production of Phenolic Acids in Salvia miltiorrhiza
Source: Int J Mol Sci. 2018 Nov 28;19(12):3788. doi: 10.3390/ijms19123788 (PMC6321555; doi:10.3390/ijms19123788)
Supplement: Supplementary file 1 [file ijms-19-03788-s001.zip › Supplementary Table 5.docx]

**Supplementary Table 5**. DEGs involved in the salvianolic acid biosynthesis

| **Gene name** | **RPKM_T0** | **Count_T0** | **RPKM_T10** | **Count_T10** | **DESeq_FDR** | **DESeq_log2FC** | **DESeq_(FDR_0.01_FC_2)_regulated** |
| --- | --- | --- | --- | --- | --- | --- | --- |
| **Phenylpropanoid pathway for phenolic acid biosynthesis** | | | | | | | |
| ***phenylalanine ammonia-lyase* (*PAL*)** | | | | | | | |
| *SMil_00012897* | 79.60265 | 2835 | 506.7335 | 17282 | 3.60E-09 | 2.617032736 | up |
| *SMil_00019885* | 101.6588 | 2272 | 428.132 | 9419 | 8.92E-07 | 2.059290005 | up |
| *SMil_00002889* | 4.849951 | 183 | 9.086741 | 397 | 0.028668989 | 1.118095145 | normal |
| ***cinnamate 4-hydroxylase* (*C4H*)** | | | | | | | |
| *SMil_00000716* | 200.5886 | 7085 | 1146.578 | 36236 | 6.79E-07 | 2.364696562 | up |
| ***4-coumarate:coenzyme A ligase* (*4CL*)** | | | | | | | |
| *SMil_00015097* | 334.4038 | 9505 | 131.2122 | 3707 | 0.086699619 | -1.354976889 | normal |
| *SMil_00024512* | 12.57114 | 447 | 31.02277 | 760 | 0.084735701 | 0.773867108 | normal |
| *SMil_00018588* | 15.77815 | 507 | 5.888527 | 203 | 0.002662979 | -1.314670623 | down |
| *SMil_00008130* | 1.963835 | 53 | 7.073498 | 191 | 0.144623608 | 1.840788154 | normal |
| *SMil_00016012* | 57.14575 | 1714 | 284.928 | 8327 | 1.62E-08 | 2.289334005 | up |
| *SMil_00021322* | 22.0385 | 722 | 9.36394 | 311 | 0.004100543 | -1.208106432 | down |
| *SMil_00004892* | 22.70293 | 814 | 32.27698 | 928 | 0.780613897 | 0.194849406 | normal |
| **Tyrosine-derived pathway forphenolic acid biosynthesis** | | | | | | | |
| ***tyrosine aminotransferase* (*TAT*)** | | | | | | | |
| *SMil_00020694* | 3.782923 | 62 | 0.965613 | 17 | 0.08672237 | -1.86388295 | normal |
| *SMil_00024925* | 290.0001 | 7067 | 1070.663 | 28849 | 1.61E-05 | 2.040040938 | up |
| *SMil_00002851* | 11.21994 | 303 | 19.89841 | 549 | 0.062131981 | 0.8639608 | normal |
| ***hydroxyphenylpyruvate reductase* (*HPPR*)** | | | | | | | |
| *SMil_00002680* | 174.1215 | 4052 | 379.357 | 9291 | 0.028847119 | 1.207651547 | normal |
| *SMil_00013707* | 18.2254 | 238 | 5.420165 | 67 | 0.001570876 | -1.807150411 | down |
| *SMil_00013867* | 5.301757 | 189 | 2.890251 | 105 | 0.242509646 | -0.841838743 | normal |
| *SMil_00026814* | 30.07065 | 101 | 87.5098 | 286 | 0.004338883 | 1.513133095 | up |
| ***rosmarinic acid synthase* (*RAS*)** | | | | | | | |
| *SMil_00008378* | 4.02347 | 73 | 4.765385 | 85 | 0.885512984 | 0.215814026 | normal |
| *SMil_00025190* | 350.889 | 10525 | 1207.975 | 36686 | 0.000237129 | 1.811942426 | up |
| *SMil_00018429* | 6.705137 | 176 | 80.75095 | 2118 | 4.73E-16 | 3.602945337 | up |
| *SMil_00025059* | 5.67925 | 117 | 9.09401 | 184 | 0.399332059 | 0.65594725 | normal |
| *SMil_00016492* | 1.095215 | 21 | 8.41038 | 158 | 0.023237555 | 2.925893705 | normal |
| ***cytochrome P450 monooxygenase CS3`H*(*CYP98A14*)** | | | | | | | |
| *SMil_00026146* | 15.0858 | 391 | 59.40725 | 1599 | 1.51E-08 | 2.038862742 | up |
