# Supplementary material for: Molecular Characterization and Overexpression of SmJMT Increases the Production of Phenolic Acids in Salvia miltiorrhiza
Source: Int J Mol Sci. 2018 Nov 28;19(12):3788. doi: 10.3390/ijms19123788 (PMC6321555; doi:10.3390/ijms19123788)
Supplement: Supplementary file 1 [file ijms-19-03788-s001.zip › Supplementary Table 6.docx]

**Supplementary Table 6.** Primer pairs used in the paper

| **Gene** | **Primer name** | **Sequence(5'-3')** | Note |
| --- | --- | --- | --- |
| *SmJMT* | *SmJMT*-F | CACAGAATTCAAGGATTCC | Molecular cloning (*SmJMT*) |
|  | *SmJMT*-R | CTAAAATTTCCTTGTGAGAG |  |
| pDONR207-*SmJMT* | pDONR207-*SmJMT*-F | GGGGACAAGTTTGTACAAAAAAGCAGGCTTCCACAGAATTCAAGGATTCC | Entry Vector Construction |
|  | pDONR207-*SmJMT*-R | GGGGACCACTTTGTACAAGAAAGCTGGGTCCTAAAATTTCCTTGTGAGAG |  |
| pEarleyGate202-35S | pEarleyGate202-35S - F | AAAGGCCCAGCAGTGATC | Transformant selection |
|  | pEarleyGate202-35S - R | GAAGGATAGTGGGATTGTGC |  |
| *SMil_00017556-* RT | *SMil_00017556-* RTF | GTCGTTGGCGGACTTCAACC | qRT-PCR (*SMil_00017556*) |
|  | *SMil_00017556-* RTR | CCGTAGAAGGAACCCGGCAT |  |
| *SMil_00019885-* RT | *SMil_00019885* - RTF | GATAGCGGAGTGCAGGTCGTAC | qRT-PCR (*SMil_00019885*) |
|  | *SMil_00019885*- RTR | CGAACTAGCAGATTGGCAGAGG |  |
| *SMil_00000716-* RT | *SMil_00000716-* RTF | CCAGGAGTCCAAATAACAGAGCCG | qRT-PCR (*SMil_00000716*) |
|  | *SMil_00000716-* RTR | GCCACCAAGCGTTCACCAAGAT |  |
| *SMil_00016012-* RT | *SMil_00016012 -* RTF | GTCGACGGCGACAACCCTAA | qRT-PCR (*SMil_00016012*) |
|  | *SMil_00016012 -* RTR | AGCAGCGAGCCGATCTCAAA |  |
| *SMil_00024925-* RT | *SMil_00024925*- RTF | CGAGCAGGGATGGGAGGTTG | qRT-PCR (*SMil_00024925*) |
|  | *SMil_00024925 -* RTR | GCCTCTTGGCTGTCTCAGCA |  |
| *SMil_00002680-* RT | *SMil_00002680* - RTF | TGACTCCAGAAACAACCCACATT | qRT-PCR (*SMil_00002680*) |
|  | *SMil_00002680 -* RTR | CCCAGACGACCCTCCACAAG |  |
| *SMil_00025190-* RT | *SMil_00025190*- RTF | CCAAAGTCAATTATGCCAAGGG | qRT-PCR (*SMil_00025190*) |
|  | *SMil_00025190* - RTR | GTCGGATAGGTGGTGCTCGT |  |
| *SMil_00026146-* RT | *SMil_00026146*- RTF | CCAATCCTACGGCCCGATCC | qRT-PCR (*SMil_00026146*) |
|  | *SMil_00026146* RTR | GCCGTCTCTGCTGAGCTTGA |  |
| *SMil_00004108-* RT | *SMil_00004108*- RTF | TTCTTCCTCCGCTACGACTC | qRT-PCR (*SMil_00004108*) |
|  | *SMil_00004108* RTR | CGTTTGAGCGACGTTACAGT |  |
| *Smβ-actin* | *Smβ-actin*- RTF | AGGAACCACCGATCCAGACA | Inner reference |
|  | *Smβ-actin*- RTR | GGTGCCCTGAGGTCCTGTT |  |
