# Supplementary material for: Molecular Characterization and Overexpression of SmJMT Increases the Production of Phenolic Acids in Salvia miltiorrhiza
Source: Int J Mol Sci. 2018 Nov 28;19(12):3788. doi: 10.3390/ijms19123788 (PMC6321555; doi:10.3390/ijms19123788)
Supplement: Supplementary file 1 [file ijms-19-03788-s001.zip › Supplementary Table 7.docx]

**Supplementary Table 7.** List of the JMT from different species

| **No** | **Species** | ***Name*** | **GenBank accession** |
| --- | --- | --- | --- |
| 1 | *Erythranthe guttata* | Jasmonate O-methyltransferase-like | XP_012846912 |
| 2 | *Sesamum indicum* | jasmonate O-methyltransferase | XP_011083897 |
| 3 | *Durio zibethinus* | Jasmonate O-methyltransferase-like | XP_022752170 |
| 4 | *Gossypium hirsutum* | Jasmonate O-methyltransferase-like | XP_016749084 |
| 5 | *Manihot esculenta* | jasmonate O-methyltransferase | XP_021612254 |
| 6 | *Theobroma cacao* | Jasmonic acid carboxyl methyltransferase | EOY14825 |
| 7 | *Prunus mume* | jasmonate O-methyltransferase-like | XP_008220839 |
| 8 | *Jatropha curcas* | jasmonate O-methyltransferase | XP_012071905 |
| 9 | *Ricinus communis* | jasmonate O-methyltransferase | XP_002510424 |
| 10 | *Capsicum chinense* | Jasmonate O-methyltransferase | PHT98846 |
| 11 | *Brassica napus* | jasmonate O-methyltransferase | XP_013713989 |
| 12 | *Malus domestica* | jasmonate O-methyltransferase-like | XP_008388031 |
| 13 | *Hevea brasiliensis* | jasmonate O-methyltransferase | XP_021663638 |
| 14 | *Citrus sinensis* | jasmonate O-methyltransferase-like | XP_006478462 |
| 15 | *Helianthus annuus* | jasmonate O-methyltransferase-like | XP_022000380 |
| 16 | *Citrus clementina* | jasmonate O-methyltransferase-like | XP_024042875 |
| 17 | *Prunus avium* | jasmonate O-methyltransferase-like | XP_021831606 |
| 18 | *Pyrus x bretschneideri* | jasmonate O-methyltransferase-like | XP_009336030 |
| 19 | *Prunus persica* | jasmonate O-methyltransferase | XP_007223087 |
| 20 | *Solanum tuberosum* | jasmonate O-methyltransferase | XP_006341965 |
| 21 | *Brassica rapa* | jasmonate O-methyltransferase-like | XP_009103381 |
| 22 | *Nicotiana attenuata* | jasmonate O-methyltransferase | XP_019267341 |
| 23 | *Brassica oleracea* | jasmonate O-methyltransferase-like | XP_013584159 |
| 24 | *Lactuca sativa* | jasmonate O-methyltransferase | XP_023767190 |
| 25 | *Solanum lycopersicum* | jasmonate O-methyltransferase | XP_004238275 |
| 26 | *Fragaria vesca* | jasmonate O-methyltransferase-like | XP_004291852 |
| 27 | *Salvia_miltiorrhiza* | Jasmonic acid carboxyl methyltransferase | MH136806 |
| 28 | *Arabidopsis thaliana* | jasmonic acid carboxyl methyltransferase | AEE29876 |
