# Supplementary figures and images for: Molecular Characterization and Overexpression of SmJMT Increases the Production of Phenolic Acids in Salvia miltiorrhiza
Source: Int J Mol Sci. 2018 Nov 28;19(12):3788. doi: 10.3390/ijms19123788 (PMC6321555; doi:10.3390/ijms19123788)

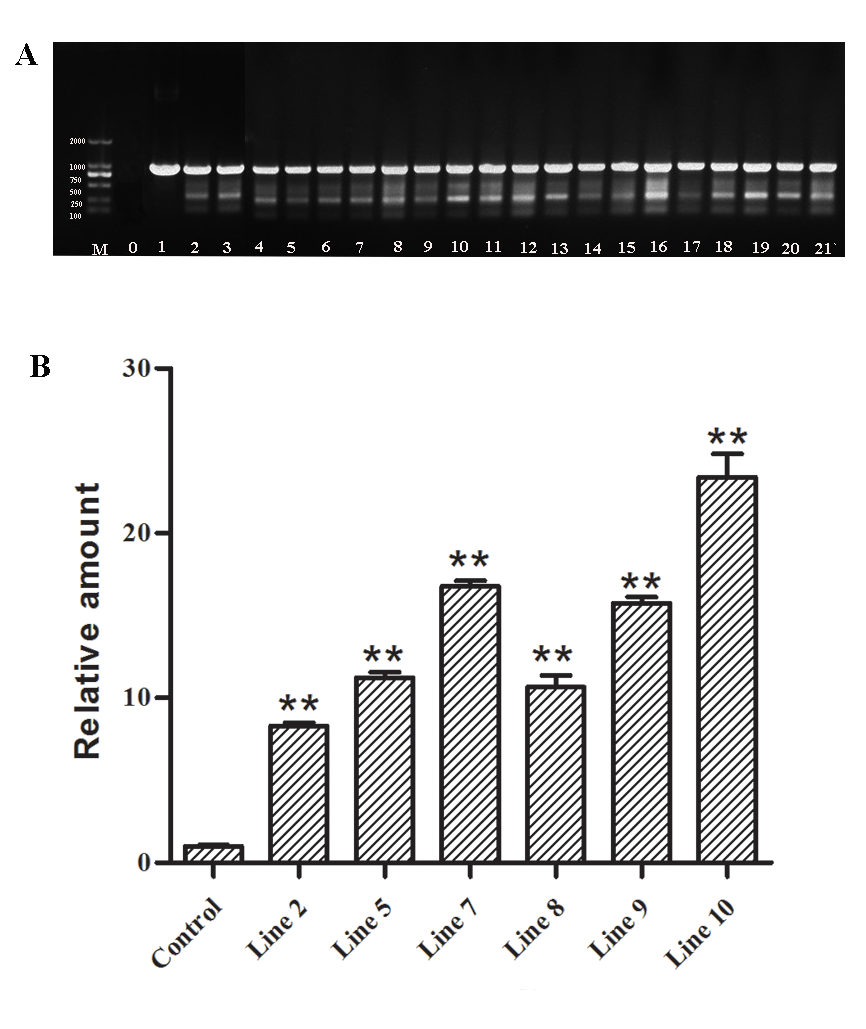

Supplement: Supplementary file 1 [file ijms-19-03788-s001.zip › Supplementary Figure 1.tif]

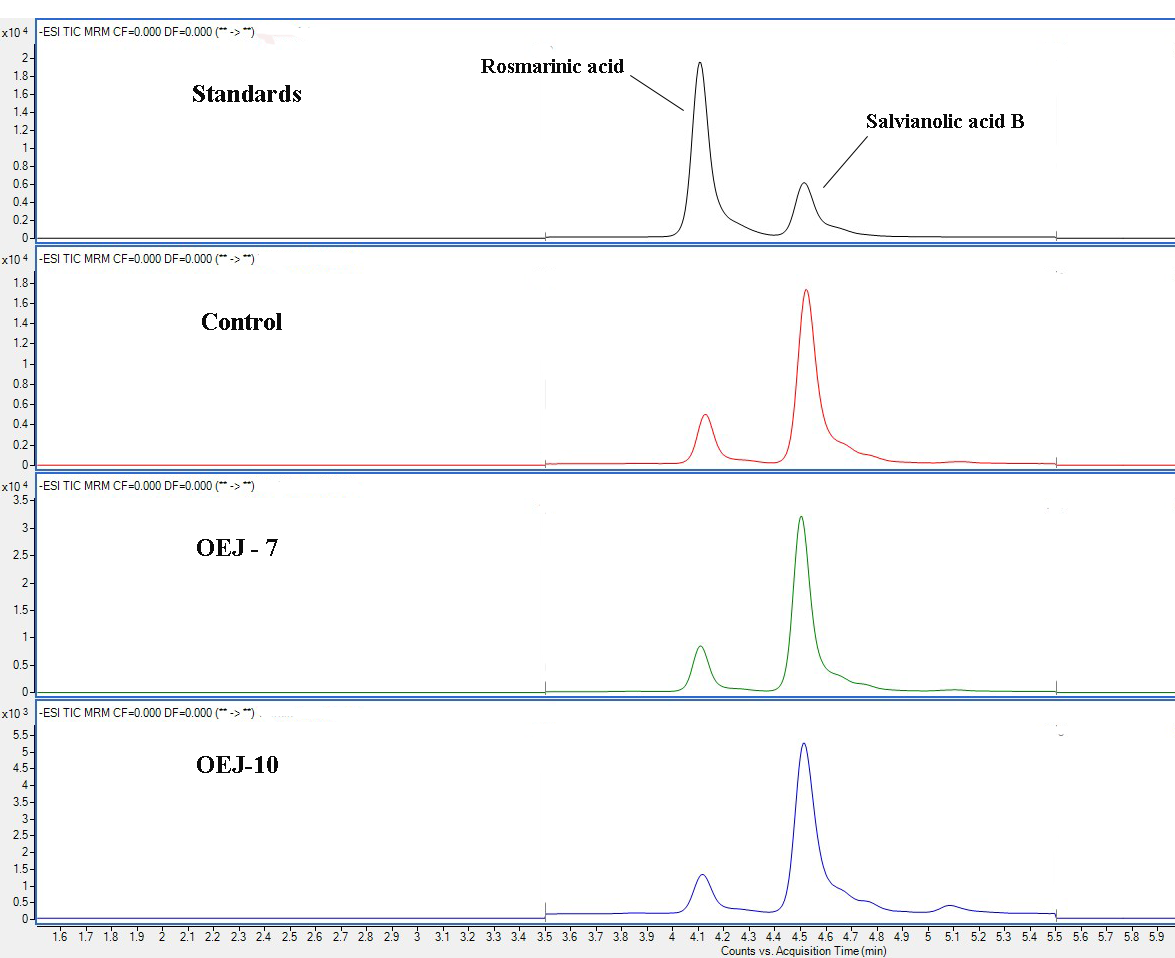

Supplement: Supplementary file 1 [file ijms-19-03788-s001.zip › Supplementary Figure 2.tif]

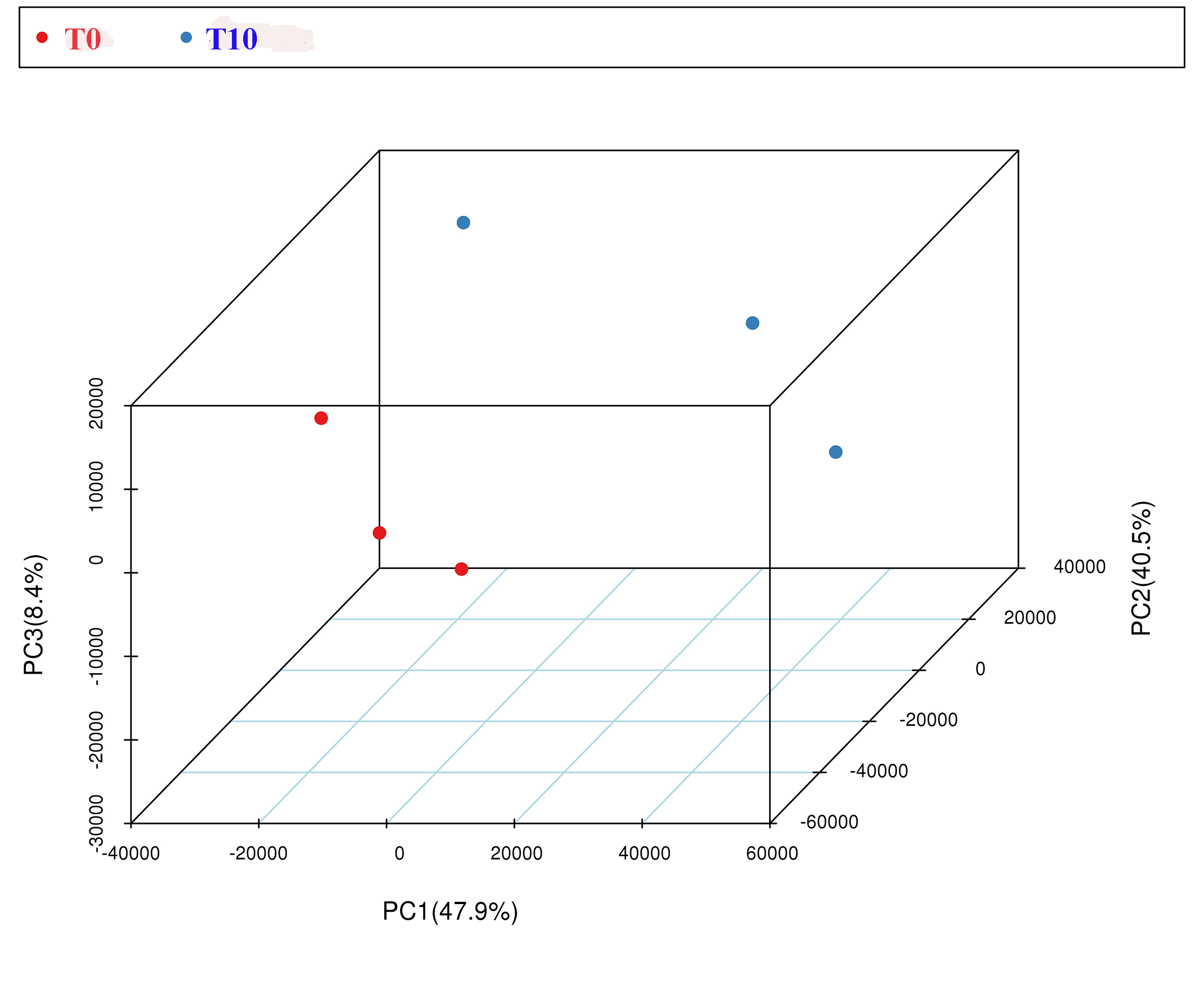

Supplement: Supplementary file 1 [file ijms-19-03788-s001.zip › Supplementary Figure 3.tif]

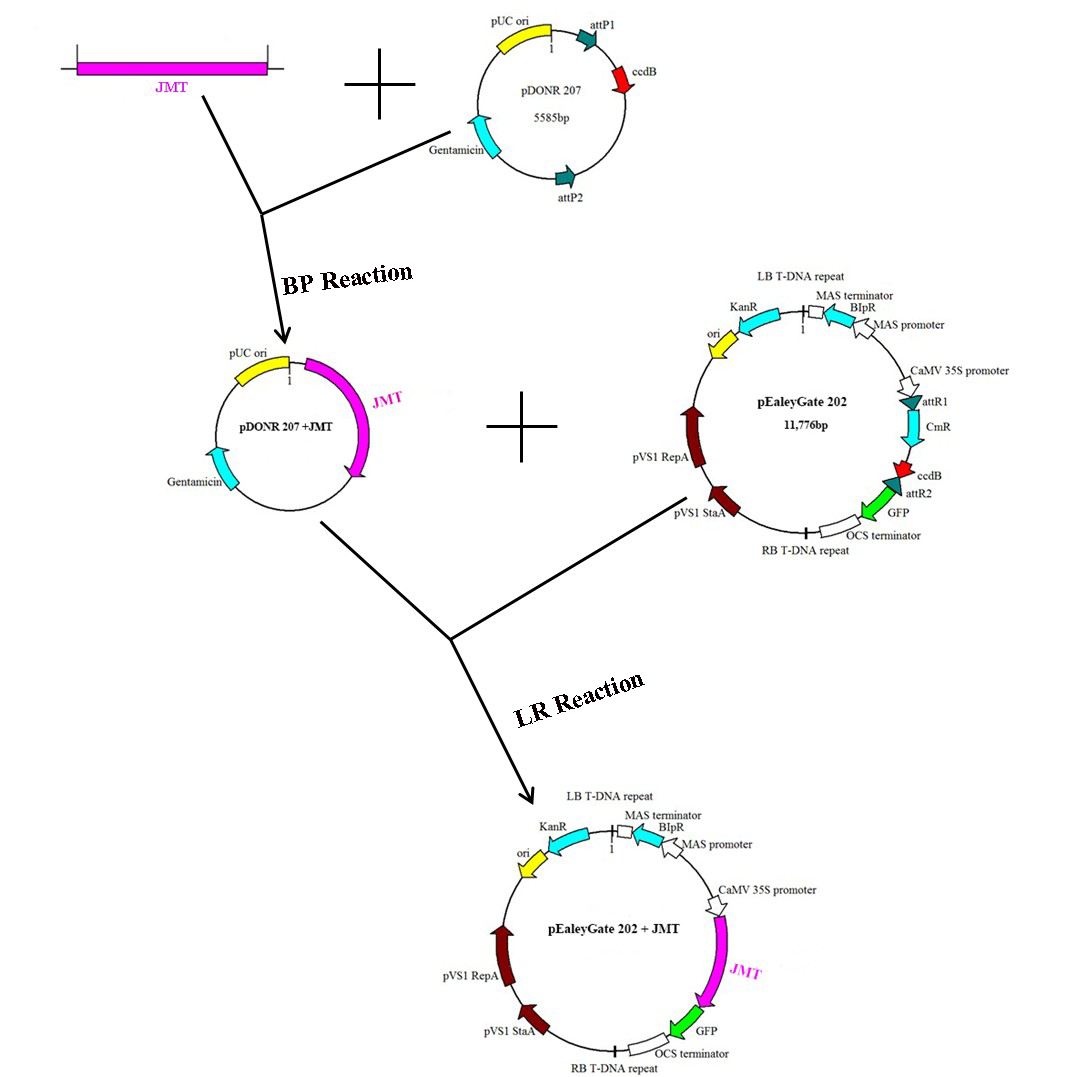

Supplement: Supplementary file 1 [file ijms-19-03788-s001.zip › Supplementary Figure 4.tif]
